# Comparative evaluation of spontaneous breathing trial techniques for ventilator weaning: a bench study

**DOI:** 10.1186/s40635-025-00788-y

**Published:** 2025-08-05

**Authors:** Guillaume Fossat, Roberto Martínez Alejos, Emeline Fresnel, Mai-Anh Nay, Clément Medrinal, Marius Lebret

**Affiliations:** 1 Univ Rouen Normandie, GRHVN UR3830, Institute for Research and Innovation in Biomedicine (IRIB), 76000 Rouen, France; 2 Intensive Care Unit Department, ORLEANS University Hospital, 45067 Orléans, France; 3 Kernel Biomedical, 18 Rue Marie Curie Bâtiment ANIDER, 76000 Rouen, France; 4 Montpellier University Training School of Physiotherapy, 34090 Montpellier, France; 5 Deusto University, 20012 San Sebastian, Spain; 6 Physiotherapy Department, Le Havre Hospital, 76600 Le Havre, France; 7 Vent’lab, Intensive Care Unit Department, Angers University Hospital, 49100 Angers, France

**Keywords:** Spontaneous breathing trial, High flow, Weaning, Work of breathing, Dead space washout, ICU

## Abstract

**Background:**

Spontaneous breathing trials (SBT) are crucial for determining when mechanically ventilated patients are ready for extubation. While pressure support (PS) and T-piece trials are commonly used, humidified high-flow (HHF) is increasingly considered, but its physiological effects remain unclear. This bench study compares T-piece, PS, and HHF modalities, evaluating their impact on work of breathing (WOB), tidal volume (Vt), total positive end-expiratory pressure (PEEPtot) and CO_2_ clearance.

**Methods:**

A 3D-printed manikin head connected to an artificial lung was used. Four SBT modalities were tested: T-piece with and without supplemental oxygen, PS at 7 cmH_2_O (PEEP 0 cmH_2_O), and HHF at 50 L/min. The tests were performed under three lung conditions (normal, obstructive, restrictive) and two respiratory drive and effort settings (normal and intense), resulting in 24 scenarios. Measurements included WOB, CO_2_ clearance, PEEPtot, and Vt.

**Results:**

T-piece and HHF50 SBTs exhibited similar effects on WOB, irrespective of the effort pattern associated with the underlying respiratory mechanics. For intense effort patterns, the CO_2_ concentration was lower with HHF than with PS, regardless of respiratory mechanics. The HHF50 SBT increased PEEPtot more than T-piece SBTs, but less than PS SBT, for all scenarios. HHF50 SBT generated lower tidal volume than T-piece and PS SBTs.

**Conclusions:**

Humidified high-flow at 50 L/min, while preserving WOB and not increasing tidal volume, may offer specific advantages, such as improved CO_2_ clearance and PEEP effect, and could be considered as a trade-off for T-piece or PS SBTs.

**Supplementary Information:**

The online version contains supplementary material available at 10.1186/s40635-025-00788-y.

## Background

Invasive mechanical ventilation is frequently employed in the intensive care unit (ICU) to support patients with respiratory failure. However, prolonged use of mechanical ventilation is associated with a higher risk of complications [1], emphasizing the importance of timely initiation of the weaning process [2]. Successful weaning, defined as the complete discontinuation of mechanical ventilation, is achieved in most cases, although failure rates range from 15 to 25% in high-risk populations [3, 4]. Weaning delays or failures are associated with increased morbidity and mortality in both ICU and hospital settings [5–7].

A key step in weaning is the spontaneous breathing trial (SBT) [2], which evaluates the ability of the patient to sustain independent breathing by reducing or eliminating ventilatory support while intubated. SBTs simulate the physiological workload following extubation [8] and typically last 30 to 120 min, during which clinical tolerance is assessed [1]. The choice of SBT technique, such as the T-piece trials or pressure support ventilation, can influence the outcomes [9, 10]. Observational data suggest that failure of the initial SBT correlates with worse clinical outcomes [11], underscoring the need for effective methods to optimize weaning and avoid premature or delayed extubation.

A novel SBT technique employs humidified high-flow (HHF) through a specialized endotracheal tube (ETT) connector [12, 13]. Preliminary data suggest potential benefits of HHF for SBT, including reduced work of breathing and enhanced positive end-expiratory pressure (PEEP) effect [14], but these findings are based on a bench model that did not embed active humidification and carbon dioxide (CO_2_) washout measurement [15]. Moreover, comparisons of HHF for SBT with other established techniques, such as pressure support or T-piece trials, remain scarce.

To address these gaps, we conducted a comparative evaluation of HHF, T-piece, and pressure support as SBT under varying respiratory conditions. Our study assessed key physiological parameters, including work of breathing (WOB), total PEEP (PEEPtot), tidal volume (Vt) and CO_2_ washout, to better understand the mechanisms underlying HHF SBT and its potential role in ICU weaning protocols.

## Methods

### Experimental setup

We utilized a 3D-printed manikin head (Kernel Biomedical, France) replicating the upper airway anatomy down to the trachea, as previously described [16, 17]. A size 7.5 ETT (internal diameter: 7.5 mm, outer diameter: 10.3 mm, total length: 30 cm) was introduced through the mouth to the trachea, with the dental marker positioned at 23 cm, as recommended [18]. The ETT cuff was inflated to its maximum capacity to prevent leakage. The distal end of the trachea was connected to an artificial lung (ASL 5000, IngMar Medical, USA) via a 22-mm-diameter breathing circuit. A protective balloon (AGEC, IngMar Medical, USA) was placed between the trachea and the artificial lung to prevent moisture from affecting the internal components of the mechanical lung.

**Fig. 1:**
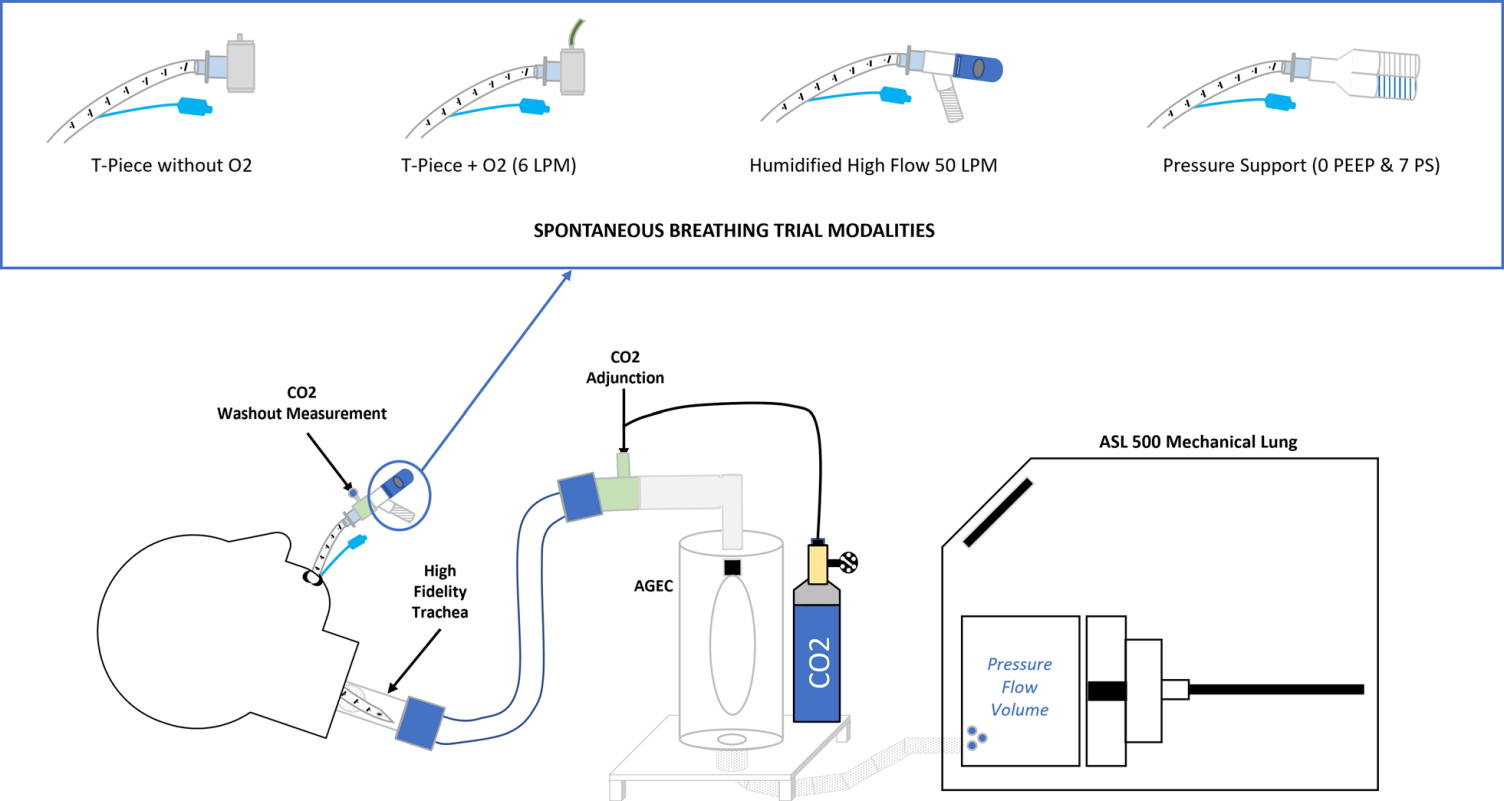
Experimental bench model and SBT conditions. *Blue points* indicate locations of measures. *SBT* spontaneous breathing trial, *LPM* liters per minute, *PEEP* positive end-expiratory pressure, *PS* pressure support, *CO*_*2*_ carbon dioxide, *AGEC* protective balloon

The total length of the breathing circuit between the manikin head and the artificial lung was 150 cm. Compliances of the protective balloon and other components were considered by entering compensation parameters in the software governing the mechanical lung (ASL 5000 software, version 3.6), in accordance with the manufacturer’s instructions. CO_2_ was introduced into the circuit just proximal to the protective balloon with a continuous flow rate of 0.2 L/min to simulate CO_2_ production during exhalation. The complete experimental setup is illustrated in Fig. 1 and shown in supplementary material (Fig. S1).

### Spontaneous breathing trial protocol

Four SBT modalities were evaluated:

1. T-piece (Trach-Vent+, Teleflex Medical, Ireland) without O_2_.
2. T-piece (Trach-Vent+, Teleflex Medical, Ireland) with an oxygen flow rate of 6 L/min (T-PieceO_2_).
3. Pressure support (PS) of 7 cmH_2_O without PEEP (0 cmH_2_O) (Servo-U, Getinge, Gothenburg, Sweden), using a heated and humidified circuit (MR850, Fisher & Paykel, Auckland, New Zealand) set at 37 °C.
4. Humidified high-flow (HHF) with a circuit temperature of 37 °C and a flow of 50 L/min (Airvo3, Fisher & Paykel, Auckland, New Zealand). This modality will be named HHF50.

### Pulmonary, drive and effort patterns

We simulated three specific pulmonary conditions by adjusting the parameters of airway resistance and thoraco-pulmonary compliance, as well as the parameters of respiratory drive and effort patterns (hereafter simply referred to as *effort pattern*) with P0.1, respiratory rate (RR) and esophageal pressure (Pes), as used in another bench study [14, 19] (Table S1). Each combination of pulmonary condition and *effort pattern* produced a unique inspiratory tidal volume/minute ventilation setup (Table S2) that was tested across all four SBT modalities, yielding 24 unique scenarios. The inspiratory-to-expiratory ratio (I:E) was set at 1:2, resulting in a 1-s inspiration phase and a 0.7-s expiration phase for the normal and intense effort patterns (P0.1 setting of 3.5 and 6.0 cmH_2_O, respectively). For each scenario, 50 respiratory cycles were analyzed.

The measured outcomes were:

1. Work of breathing (WOB) (J/L).
2. CO_2_ concentration (ppm).
3. PEEPtot (cmH_2_O).
4. Tidal volume (Vt) (mL).

### Statistical analysis

Quantitative data were expressed as median (interquartile range). Normality of the distribution was assessed using the Shapiro–Wilk test. We use the Kruskal–Wallis with the Dunn’s post hoc tests for multiple comparisons. Tests were two-tailed, and a value of *p* < 0.05 was considered statistically different. Statistical analysis was performed using GraphPad Prism 10.4.1 (GraphPad Software, USA).

## Results

### WOB

**Fig. 2:**
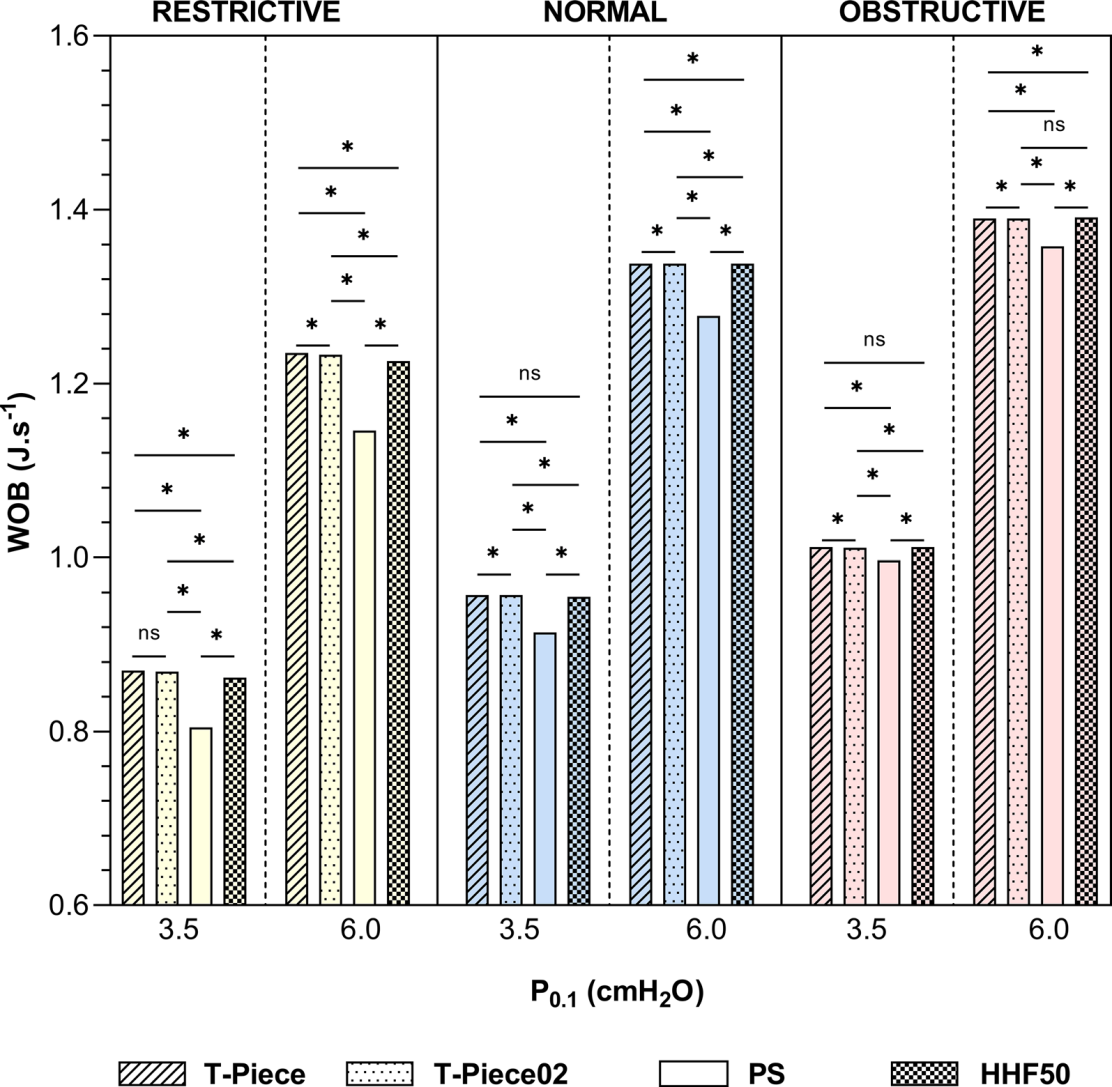
Work of breathing according to each spontaneous breathing trial scenario. * indicates statistical significance difference (*p* < 0.001) using Kruskal–Wallis tests and pairwise comparison using Dunn’s post hoc tests. Median absolute values and interquartile range are presented in Table S3. *WOB* work of breathing, *J L*^*−1*^ Joule per liter, *cmH*_*2*_*O* centimeter of water, *PS* pressure support, *HHF50* humidified high-flow at 50 L/min, *NS* non-significant

WOB was lower with PS SBT under all conditions (*p* < 0.001). T-piece, T-pieceO_2_, and HHF50 exhibited similar effects on WOB for normal effort pattern. Independent of the SBT modality, obstructive respiratory mechanics were associated with higher WOB compared to both normal and restrictive mechanics (*p* < 0.001). Additionally, normal mechanics were linked to greater WOB than restrictive mechanics (*p* < 0.001) (Fig. 2 and Table S3).

### *CO*_*2*_* concentration*

**Fig. 3:**
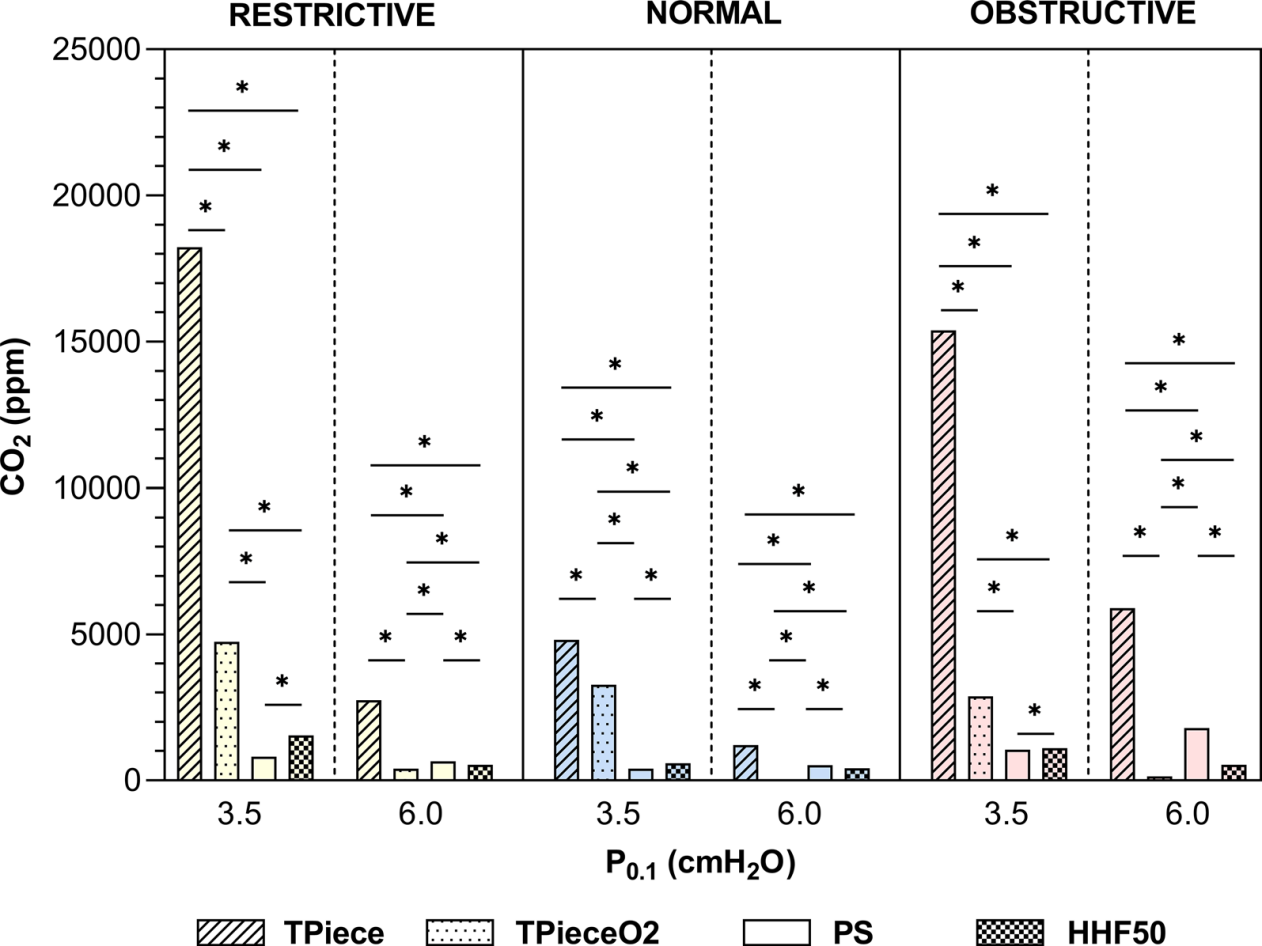
CO_2_ concentration at the end of each spontaneous breathing trial scenario. * indicates statistical significance difference (*p* < 0.001) using Kruskal–Wallis tests and pairwise comparison using Dunn’s post hoc tests. Median absolute values and interquartile range are presented in Table S4. *ppm* part per million, *cmH*_*2*_*O* centimeter of water, *PS* pressure support, *HHF50* humidified high-flow at 50 L/min

The T-piece without oxygen SBT resulted in a higher CO_2_ concentration at the end of each scenario (*p* < 0.001). For the intense effort pattern, the CO_2_ concentration was lower with HHF50 than with PS, regardless of respiratory mechanics (*p* < 0.001). The T-Piece O2 SBT lowered the CO_2_ concentration more than the HHF50 SBT for the intense effort pattern (*p* < 0.001). For the normal effort pattern, the CO_2_ concentration at the end of the SBT was most reduced during PS, followed by HHF50, and finally T-Piece O2, *p* < 0.001 for every comparison (Fig. 3 and Table S4)*.*

### PEEPtot

The pressure support SBT increased PEEPtot more than all other modalities under all conditions (*p* < 0.001). The HHF50 SBT increased PEEPtot more than T-piece SBTs for all scenarios (*p* < 0.001). Normal respiratory mechanics resulted in the highest PEEPtot values, regardless of SBT modality or effort pattern (*p* < 0.001) (Table S5).

### Tidal volume

Pressure support SBT led to the greatest improvement in tidal volume under all conditions (*p* < 0.001). HHF50 SBT generated lower tidal volume than T-Piece SBTs (*p* < 0.001) (Table S6).

## Discussion

Our bench study is the first to compare 3 SBT modalities. Our results show that PS SBT is the modality that most facilitates pulmonary bench model outcomes, while it decreases WOB, increases tidal volume, and generates a higher PEEPtot when compared to unassisted SBTs. Our results highlight that T-piece SBT is the least facilitating of the studied SBTs, except for CO_2_ clearance during intense effort. Finally, the 50 L/min humidified high-flow SBT seems to be a good trade-off for the T-piece or PS SBTs. It generates a WOB identical to that of the T-piece, while slightly improving PEEPtot and CO_2_ clearance without increasing tidal volume. This absence of variation in tidal volume during HHF50 SBT, for the same artificial lung setting, may indicate that this modality does not provide any support when compared to other SBT modalities.

SBT aimed to replicate WOB that would be supported by the patient after extubation [14]. Under these conditions, it is crucial for clinicians to use the most effective SBT method to predict the respiratory strain that the patient will experience after extubation. A reduction in WOB during SBT may hide critical markers of weaning failure [20]. Our bench seems to confirm that PS SBT is the most facilitative; this aligns with Sklar et al.’s clinical meta-analysis showing a 30% reduction in WOB with PS SBT compared to T-Piece [8]. Our model showed that HHF SBT achieves no WOB reductions when compared to PS SBT. Other bench studies reported comparable WOB between HHF and unassisted breathing modalities, using intubated SBT bench model [14] or tracheotomized bench model [17]. However, the obstructive respiratory model appeared to limit the relative improvement in WOB, suggesting that underlying respiratory pathophysiology specificities may benefit from personalized SBT.

Our results showed marked variability for CO_2_ clearance across SBT modalities. PS showed superior performance under normal effort pattern, while T-PieceO_2_ SBT appeared more effective during intense effort pattern. HHF SBT, which does not reduce WOB, also showed notable efficacy in CO_2_ clearance, outperforming PS in conditions of elevated respiratory drive and effort. To our knowledge, our study is the first to examine CO_2_ clearance during SBT. Improved CO_2_ washout may increase the availability of oxygen in the dead space [21]. In a preliminary clinical trial [12], we observed that the drop in PaO_2_ during SBT was less pronounced in the HHF SBT group compared to the T-piece O_2_ group, in at risk of weaning failure patients.

Considering PEEPtot, it may seem unexpected to observe such high values during PS-SBT. Conversely, Sameed et al. [22] have highlighted that Servo ventilators (Getinge, Gothenburg, Sweden) impose a flow by of 2 L/min. In their bench study, T-Piece was simulated using 0 cmH_2_O of PS and 0 cmH_2_O of PEEP. Under these settings, the Servo U ventilator imposes a pressure of 4.5 cm H_2_O above PEEP during the inspiratory phase. Secondly, I:E ratio and the high compliance of obstructive and normal respiratory mechanics used in our models may have resulted in an expiratory time that was too short for complete exhalation. This may result in residual intrinsic pressure. Overall, the combination of Servo-U flow by and I:E ratio used in our model could be responsible for the PEEPtot generated during pressure support mode without PEEP. The enhanced PEEPtot observed with HHF SBT aligns with prior bench studies on tracheotomized models [17, 23]. These studies demonstrated that high-flow devices could generate a low but measurable PEEP effect, albeit to a lesser extent than PS.

While SBT’s intended goal is to replicate natural respiratory mechanics, PS seems to be the modality that mostly facilitates ventilation by increasing tidal volume. While unassisted modalities (T-Piece and HHF) failed to generate comparable tidal volumes, our findings mirror previous results where high-flow devices at 40 L/min produced only modest increases in tidal volume [14, 23]. In a preliminary clinical trial [12], we observed that PaCO_2_ remained stable over the SBT’s courses, indicating that neither the T-piece nor the high-flow SBT can reduce CO_2_ by increasing ventilation.

One limitation of this bench study is that it did not assess flow rates other than 50 L/min with HHF SBT. This choice was made to reflect common clinical practices, as many ICUs use high-flow oxygen therapy delivered through ICU ventilators. Notably, several of these ventilators are unable to deliver flow rates of 60 L/min [24].

In addition to testing multiple SBTs under a wide range of conditions, we wanted to maintain active humidification with the PS and HHF modalities. This was a deliberate effort to enhance the clinical applicability of our model. Unlike passive heat and moisture exchangers, heated humidifiers achieve superior humidity and reduced dead space volume [25, 26], although they may impose additional respiratory load [27]. A recent clinical cross-over study showed that unassisted SBTs (i.e., T-Piece and Zero PEEP/Zero PS) produced the same respiratory responses as spontaneous breathing after extubation, whereas PS SBT attenuated them [28]. PS SBT appears to be the modality that most affects WOB, although its role as a predictor of extubation success remains debated. Recent clinical studies [9, 10] and subsequent meta-analyses [29, 30] indicate that the pressure support SBT allows patients to complete the SBT process more quickly and without the need for reintubation, in comparison with the T-Piece SBT but does not necessarily predict post-extubation respiratory performance.

Humidified high-flow SBT demonstrates potential advantages in improving CO_2_ clearance and PEEP effect while not impacting WOB and tidal volume. However, their clinical adoption remains limited due to insufficient robust patient data [29]. While our model incorporates elements reflective of real-world ICU practices [9, 10], it remains a bench study with inherent limitations, and caution is warranted when extrapolating these findings to clinical practice.

## Conclusions

Replicating the respiratory burden after extubation is one of the roles of SBT. Humidified high-flow at 50 L/min, while preserving WOB and not increasing tidal volume, may offer specific advantages, such as improved CO_2_ clearance and PEEP effect, and could be considered as a trade-off for T-Piece or PS SBTs. Further clinical studies are warranted to validate these observations and better define the role of HHF during SBT in weaning protocols.

## Supplementary Information

Additional file 1.

## Data Availability

Guillaume Fossat had full access to all the data in the study and takes responsibility for the integrity of the data and the accuracy of the data analysis. The study specific summary data can be obtained from the corresponding author: Guillaume Fossat, guillaume.fossat@chu-orleans.fr.

## Abbreviations

SBT: Spontaneous breathing trial
PS: Pressure support
HHF: Humidified high-flow
WOB: Work of breathing
Vt: Tidal volume
PEEP: Positive end-expiratory pressure
ICU: Intensive care unit
ETT: Endotracheal tube
cmH_2_O: Centimeter of water
mL: Milliliter
J/L: Joules per liter
ppm: Parts per million
