# Supplementary material for: Comparative evaluation of spontaneous breathing trial techniques for ventilator weaning: a bench study
Source: Intensive Care Med Exp. 2025 Aug 5;13:78. doi: 10.1186/s40635-025-00788-y (PMC12325126; doi:10.1186/s40635-025-00788-y)
Supplement: Supplementary file 1 — Additional file 1. [file 40635_2025_788_MOESM1_ESM.docx]

**Supplemental materials**

**Image S1 : Bench setup with high fidelity trachea mannequin connected to mechanical lung**


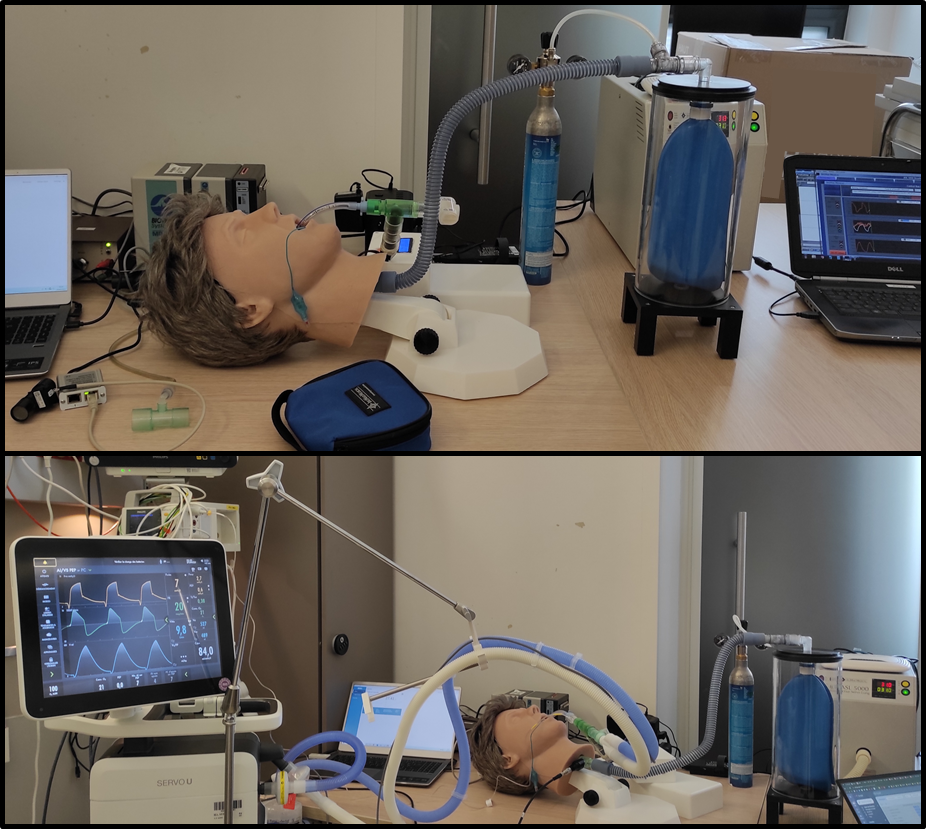

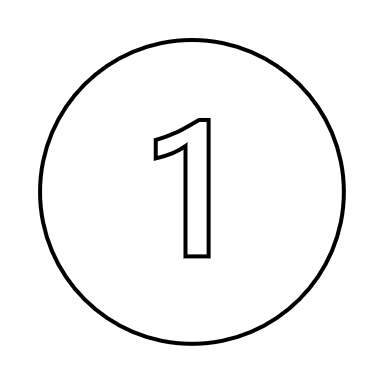

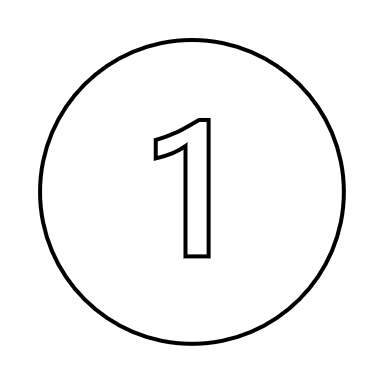

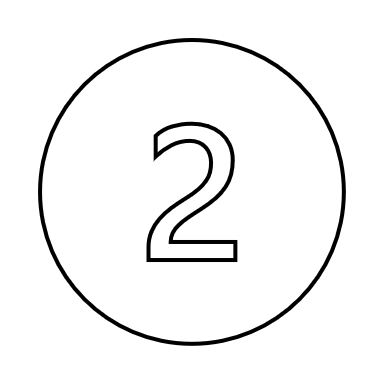

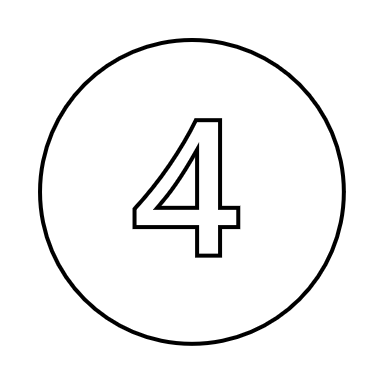

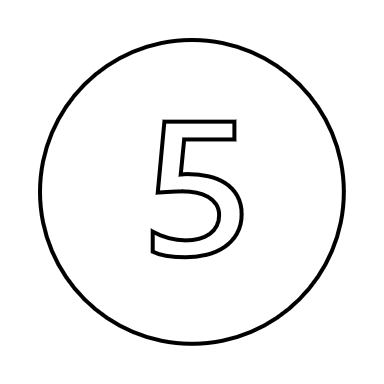

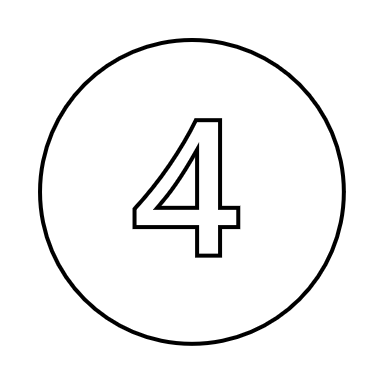

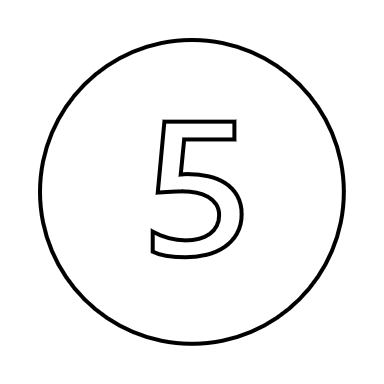

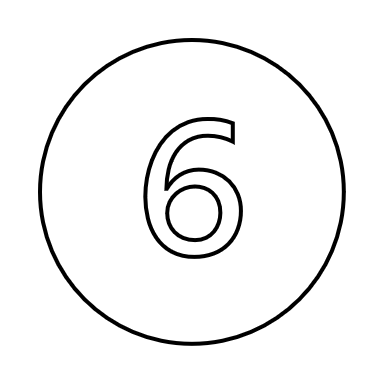

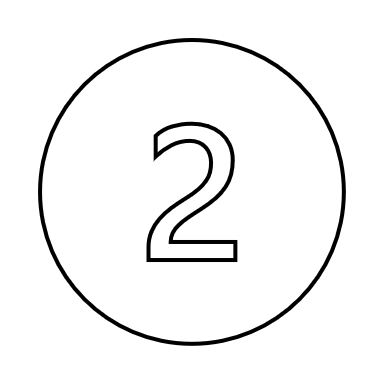

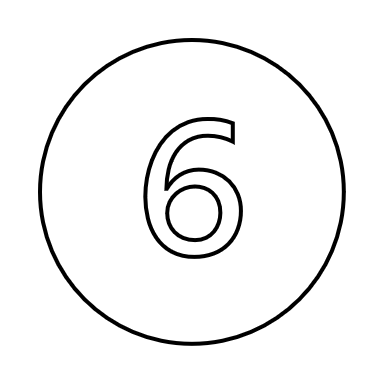

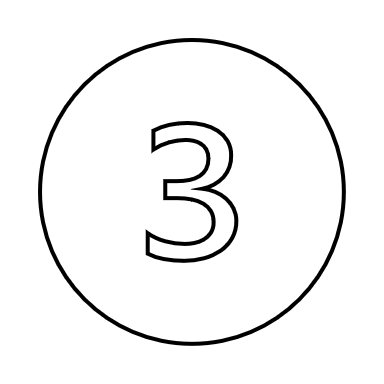


**Legend**

1. 3D-printed manikin head; 2. CO2 Adjunction in mechanical lung circuit; 3. CO2 concentration measurement at the end of endotracheal tube; 4. Mechanical lung (ASL5000, IngarMedical, USA); 5. Protective balloon (AGEC, IngarMedical, USA); 6. Spontaneous breathing trial modality connection to the endotracheal tube.

**Table S1: Simulated parameters incorporated in the ASL500**

| Respiratory mechanics | | | Respiratory drive and effort patterns | | | | |
| --- | --- | --- | --- | --- | --- | --- | --- |
|  | Resistance (cmH2O.s/L) | Compliance (ml/cmH_2_O) |  | P0.1 (cmH2O) | RR (cpm) | Pes (cmH2O) |  |
| Normal | 5 | 60 | Normal | 3.5 | 20 | -11.9 |  |
| Restrictive | 5 | 30 | Intense | 6 | 30 | -14.3 |  |
| Obstructive | 20 | 60 |  |  |  |  |  |

## Legend:

RR: Respiratory Rate; Pes: Esophageal Pressure. Simulated parameters were obtained from [1, 2]

## Table S2 : Final script applied to each spontaneous breathing trial

| Final Script | | | | |
| --- | --- | --- | --- | --- |
|  | Respiratory Drive and Effort | Respiratory  mechanics | Inspired Tidal volume (mL) | Minute ventilation (L/min) |
| 1 | Normal | Restrictive | 306 | 6.1 |
| 2 | Normal | Normal | 589 | 11.8 |
| 3 | Normal | Obstructive | 279 | 5.6 |
| 4 | Intense | Restrictive | 384 | 11.5 |
| 5 | Intense | Normal | 650 | 19.5 |
| 6 | Intense | Obstructive | 257 | 7.7 |

## Table S3: Absolutes values for work of breathing according to each scenario

|  | | WOB in J/L | | | |  |
| --- | --- | --- | --- | --- | --- | --- |
| Drive & Effort | Respiratory mechanics | T-Piece | T-PieceO2 | Pressure Support | HHF 50 | Kruskal Wallis  p-value |
| Normal | Restrictive | 0.87 [0.87 - 0.87]* | 0.87 [0.87 - 0.87]* | 0.81 [0.81 - 0.81] | 0.88 [0.88 - 0.88] | <0.001 |
|  | Normal | 0.96 [0.96 - 0.96]^†^ | 0.96 [0.96 - 0.96] | 0.91 [0.91 - 0.91] | 0.96 [0.96 - 0.96]^†^ | <0.001 |
|  | Obstructive | 1.01 [1.01 - 1.01]^†^ | 1.01 [1.01 - 1.01] | 1.00 [1.00 - 1.00] | 1.01 [1.01 - 1.01]^†^ | <0.001 |
| Intense | Restrictive | 1.24 [1.24 - 1.24] | 1.24 [1.24 - 1.24] | 1.15 [1.15 - 1.15] | 1.24 [1.24 - 1.24] | <0.001 |
|  | Normal | 1.34 [1.34 - 1.34] | 1.34 [1.34 - 1.34] | 1.30 [1.30 - 1.30] | 1.34 [1.34 - 1.34] | <0.001 |
|  | Obstructive | 1.39 [1.39 - 1.39] | 1.39 [1.39 - 1.39]^ǂ^ | 1.36 [1.36 - 1.36] | 1.39 [1.39 - 1.39]^ǂ^ | <0.001 |

## Legend:

Absolute values are presented as median and Interquartile range. Kruskal Wallis tests show statistical difference for every condition, as almost every pairwise comparison using Dunn's post hoc tests show statistical significance difference (p<0.001 in each case), we choose only to highlight non-significant results. *NS between T-Piece and T-Piece02; ^†^NS between T-Piece and HHF50; ^ǂ^NS between T-PieceO2 and HHF50.

HHF: Humidified high flow ; WOB : Work of Breathing ; J/L : Joules per liter; NS: Non significant

**Table S4: Absolute values for CO2 concentration at the end of study procedure according to each scenario**

|  | | CO2 concentration in ppm | | | |  |
| --- | --- | --- | --- | --- | --- | --- |
| Drive & Effort | Respiratory mechanics | T-Piece | T-PieceO2 | Pressure Support | HHF 50 | Kruskal Wallis  p-value |
| Normal | Restrictive | 18228 [17365 - 19091] | 4746 [4330 - 5163] | 818 [787 - 849] | 1547 [1486 - 1609] | <0.001 |
|  | Normal | 4813 [4783 - 4843] | 3277 [3104 - 3450] | 405 [403 - 406] | 583 [531 - 634] | <0.001 |
|  | Obstructive | 15384 [14869 - 15899] | 2872 [2410 - 3333] | 1053 [985 - 1121] | 1101 [1090 - 1112] | <0.001 |
| Intense | Restrictive | 2746 [2527 - 2966] | 400 [392 - 408] | 657 [626 - 688] | 530 [504 - 556] | <0.001 |
|  | Normal | 1212 [1196 - 1228] | 0 [0 - 0] | 524 [484 - 564] | 414 [405 - 423] | <0.001 |
|  | Obstructive | 5894 [5860 - 5927] | 145 [0 - 367] | 1799 [1783 - 1816] | 530 [526 - 534] | <0.001 |

**Legend:**

Absolute values are presented as mean and standard deviation. Kruskal Wallis tests show statistical difference for every condition, every pairwise comparison using Dunn's post hoc tests show statistical significance difference (p<0.001 in each case).

HHF: Humidified high flow; ppm : parts per million; PS: Pressure Support.

**Table S5: Absolute values for total positive end-expiratory pressure according to each scenario**

|  | | PEEPtot in cmH2O | | | |  |
| --- | --- | --- | --- | --- | --- | --- |
| Drive & Effort | Respiratory mechanics | T-Piece | T-PieceO2 | Pressure Support | HHF 50 | Kruskal Wallis  p-value |
| Normal | Restrictive | -0.06 [-0.06 - -0.05] | 0.03 [0.03 - 0.03] | 0.91 [0.91 - 0.92] | 0.64 [0.62 - 0.65] | <0.001 |
|  | Normal | 0.28 [0.27 - 0.29] | 0.37 [0.37 - 0.38] | 2.01 [2.01 - 2.02] | 1.07 [1.05 - 1.08] | <0.001 |
|  | Obstructive | 0.29 [0.29 - 0.3] | 0.4 [0.39 - 0.4] | 1.83 [1.82 - 1.84] | 1.04 [1.03 - 1.05] | <0.001 |
| Intense | Restrictive | 0.53 [0.52 - 0.53] | 0.66 [0.65 - 0.66] | 2.61 [2.6 - 2.61] | 1.5 [1.5 - 1.51] | <0.001 |
|  | Normal | 2.49 [2.49 - 2.5] | 2.64 [2.63 - 2.65] | 5.87 [5.86 - 5.88] | 3.32 [3.31 - 3.33] | <0.001 |
|  | Obstructive | 1.13 [1.13 - 1.14] | 1.24 [1.24 - 1.25] | 3.42 [3.42 - 3.43] | 1.94 [1.94 - 1.95] | <0.001 |

**Legend:**

Absolute values are presented as mean and standard deviation. Kruskal Wallis tests show statistical difference for every condition, every pairwise comparison using Dunn's post hoc tests show statistical significance difference (p<0.001 in each case).

PEEPtot: Total Positive End-Expiratory Pressure; HHF: Humidified high flow.

**Table S6: Absolute values for tidal volume according to each scenario**

|  | | Tidal Volume in mL | | | |  |
| --- | --- | --- | --- | --- | --- | --- |
| Drive & Effort | Respiratory mechanics | T-Piece | T-PieceO2 | Pressure Support | HHF 50 | Kruskal Wallis  p-value |
| Normal | Restrictive | 298 [298 - 298] | 297 [297 - 297] | 451 [450 - 451] | 289 [288 - 289] | <0.001 |
|  | Normal | 470 [470 - 470] | 467 [467 - 467] | 639 [638 - 639] | 445 [444 - 445] | <0.001 |
|  | Obstructive | 245 [245 - 245] | 244 [244 - 244] | 360 [360 - 360] | 236 [236 - 237] | <0.001 |
| Intense | Restrictive | 346 [345 - 346] | 344 [344 - 344] | 448 [448 - 449] | 330 [330 - 331] | <0.001 |
|  | Normal | 435 [435 - 435] | 430 [430 - 430] | 491 [490 - 491] | 416 [416 - 417] | <0.001 |
|  | Obstructive | 219 [219 - 219] | 217 [217 - 217] | 271 [270 - 271] | 215 [214 - 215] | <0.001 |

**Legend:**

Absolute values are presented as mean and standard deviation. Kruskal Wallis tests show statistical difference for every condition, every pairwise comparison using Dunn's post hoc tests show statistical significance difference (p<0.001 in each case).

mL: milliliters ; HHF : Humidified high flow.

References

1. Arnal J-M, Garnero A, Saoli M, Chatburn RL (2018) Parameters for Simulation of Adult Subjects During Mechanical Ventilation. Respir Care 63:158–168. https://doi.org/10.4187/respcare.05775

2. Rigault G, Guérin C, Sigaud F, et al (2023) Bench Study of a Spontaneous Breathing Trial with Different Modalities. Respir Care 68:760–766. https://doi.org/10.4187/respcare.10494
